# A Secondary Metabolite Secreted by *Penicillium citrinum* Is Able to Enhance *Parastagonospora nodorum* Sensitivity to Tebuconazole and Azoxystrobin

**DOI:** 10.3389/ffunb.2022.889547

**Published:** 2022-07-07

**Authors:** Maksim Kartashov, Tatiana Voinova, Larisa Shcherbakova, Lenara Arslanova, Kseniya Chudakova, Vitaly Dzhavakhiya

**Affiliations:** ^1^ Department of Molecular Biology, All-Russian Research Institute of Phytopathology, Moscow reg., Russia; ^2^ Laboratory of Physiological Plant Pathology, All-Russian Research Institute of Phytopathology, Moscow reg., Russia

**Keywords:** chemosensitization to agricultural fungicides, 6-demethylmevinolin, tebuconazole, azoxystrobin, *Parastagonospora nodorum*, fungicide-resistant strains

## Abstract

*Parastagonospora nodorum* causes glume and leaf blotch of wheat, a harmful disease resulting in serious losses in grain yield. In many countries including Russia, fungicidal formulations based on triazoles and on triazoles combined with strobilurins are used to control this fungus. However, their prolonged application may promote the selection of fungicide-resistant strains of *P. nodorum* leading to significant attenuation or even loss of fungicidal effect. Chemosensitization of plant pathogenic fungi with natural compounds represents a promising strategy for mitigating fungicide resistance and other negative impacts of fungicides. In this work, we applied a chemosensitization approach towards *P. nodorum* strains non-resistant or resistant to tebuconazole or azoxystrobin using 6-demethylmevinolin (6-DMM), a metabolite of *Penicillium citrinum*. The resistant strains were obtained by the mutagenesis and subsequent culturing on agar media incorporated with increasing doses of Folicur^®^ EC 250 (i.e., tebuconazole) or Quadris^®^ SC 250 (i.e., azoxystrobin). Test strains m8-4 and kd-18, most resistant to tebuconazole and azoxystrobin, respectively, were selected for sensitization experiments. These experiments demonstrated that combining 6-DMM with Folicur^®^ enhanced fungicidal effectiveness *in vitro* and *in vivo* in addition to attenuating the resistance of *P. nodorum* to tebuconazole *in vitro.* 6-DMM was also found to augment Quadris^®^ efficacy towards kd-18 when applied on detached wheat leaves inoculated with this strain. Experiments on *P. nodorum* sensitization under greenhouse conditions included preventive (applying test compounds simultaneously with inoculation) or post-inoculation spraying of wheat seedlings with 6-DMM together with Folicur^®^ at dose rates (DR) amounting to 10% and 20% of DR recommended for field application (RDR). Combined treatments were run in parallel with using the same DR of the fungicide and sensitizer, alone. A synergistic effect was observed in both preventive and post-inoculation treatments, when the sensitizer was co-applied with the fungicide at 10% of the RDR. In this case, disease reduction significantly exceeded the protective effect of Folicur^®^ at 10% or 20% of the RDR, alone, and also a calculated additive effect. Collectively, our findings suggest that 6-DMM is promising as a putative component for formulations with triazole and strobilurin fungicides. Such new formulations would improve fungicide efficacy and, potentially, lower rates of fungicides needed for plant pathogen control.

## Introduction

*Parastagonospora nodorum* (Berk.) is a necrotrophic pathogen causing glume blotch of wheat (*Triticum aestivum* L), also known as Septoria nodorum blotch (SNB) (Solomon et al., 2006). This fungus attacks not only the ear glumes directly, but also wheat leaves and culms during plant vegetation (Stone et al., 2004). In addition to wheat, *P. nodorum* can also infect other crops, e.g., barley (*Hordeum vulgare* L.) and wild cereal grasses (Mehra et al., 2019). During the course of disease development, chloroses, which appear on the leaf surfaces several days after infection by fungal spores, can develop into lesions covering part or almost the entire leaf. Diseased plants undergo reduced photosynthetic capacity and poor growth, and produce shortened ears containing shriveled and discolored grain (Sylvester et al., 2018; Mehra et al., 2018). Spores from diseased plants can be dispersed by wind or rain and contribute to infection of other plants (Sommerhalder et al., 2011).

*P. nodorum* is reportedly an extremely harmful fungal pathogen, responsible for considerable yield losses of spring and winter wheat throughout the world (Murray and Brennan, 2009; Downie et al., 2018; Boots et al., 2019; Hafez et al., 2020). For years, beginning in the 1990s (Sanin et al., 2010), this fungus became of utmost concern in the non-Chernozem zone, one of the main wheat-producing regions of Russia (Kolomiets et al., 2015; Kolomiets et al., 2017; Bakulina et al., 2020; Toropova et al., 2020).

Different approaches and agricultural practices, including seed and foliar fungicidal treatments, cereal rotation with non-host plants, tillage, and plant residue management as well as breeding for disease resistance, are being developed to combat this pathogen (Stover et al., 1996; Solomon et al., 2006; Downie et al., 2021). In addition, host–pathogen interactions are actively studied to provide progress in developing new resistant or tolerant cultivars (Hafez et al., 2020). At least eight proteinaceous effectors and nine host sensitivity genes have been already identified in *P. nodorum* isolates and wheat plants, respectively (Duba et al., 2018). In spite of this findings, breeding for resistance to glume blotch in wheat as well as technologies based on molecular plant–microbe interactions remain complicated and of limited success (Solomon et al., 2006; Francki, 2013; Downie et al., 2021), leading to a continued dependency on fungicides.

The most common wheat varieties bred in Russia and other countries are quite susceptible to SNB (Aguilar et al., 2005; Sanin et al., 2010; Downie et al., 2021). For instance, among 23 cultivars (10 from five regions of the Russian Federation and 13 from eight other countries) tested under field conditions, no cultivars with complete resistance to glume blotch were found (Toropova et al., 2019; Toropova et al., 2020). Thus, to date, one of the most reliable and affordable ways to combat *P. nodorum* is timely use of commercial fungicides at correct doses for seed and foliar treatments (Solomon et al., 2006; Toropova et al., 2019; Bakulina et al., 2020).

Various fungicidal formulations containing demethylation inhibitors (DMIs), quinone outside inhibitors (QoIs), and other active ingredients [e.g., succinate dehydrogenase inhibitors (SDHIs)] are available on the agricultural market for control of *P. nodorum*. Most are based on triazoles belonging to DMIs (such as tebuconazole, propiconazole, difenoconazole, and cyproconazole) singularly or in mixtures with QoIs (such as strobilurins, e.g., with azoxystrobin), to complement the effect of triazoles (Sylvester and Kleczewski, 2018; State Catalogue of Pesticides and Agrochemicals, 2021). In particular, Folicur^®^ EC 250, a formulation containing 25% tebuconazole as the active ingredient, is extensively used in Russia and other countries for foliar treatments for effective protection of wheat against SNB and other diseases, including Fusarium head blight, during the growing season (White, 2021). However, continued application of triazoles and strobilurins, compounds of medium to high risk for promoting development of resistance in plant pathogens (FRAC, 2021), may result in unwanted selection of fungicide-resistant strains of *P. nodorum*. Indeed, mutant strains tolerant to triazoles and strobilurins have already been identified in a number of countries where fungicide resistance is routinely monitored (Blixt et al., 2009; Pereira et al., 2020; Kaur et al., 2021). Lack of monitoring *P. nodorum* resistance to fungicides in Russia has impeded the ability to obtain a clear picture of the current state of affairs regarding this issue. However, prolonged application of triazoles and strobilurin-combined triazole formulations on an industrial scale indicates a likelihood of forthcoming appearance, or perhaps, already existing strains of the pathogen having reduced sensitivity to these antifungal agents.

One of the advanced approaches currently being developed to enhance the effect of fungicides and to reduce the impact of resistance challenge in the field of crop protection is increasing the sensitivity of plant pathogenic fungi to fungicides by environmentally safe compounds, i.e., pathogen chemosensitization (Campbell et al., 2012; Dzhavakhiya et al., 2012). Accordingly, the main goal of our study was to determine if chemosensitization using a natural product could improve the fungicidal effects of these commercial fungicides towards *P. nodorum*, including against strains resistant to tebuconazole or azoxystrobin. Since tebuconazole is the active ingredient of numerous highly effective one-component formulations, we focused special attention to sensitization of the pathogen to this fungicide. In our prior experiments, we showed that a microbial metabolite, 6-demethylmevinolin (6-DMM), was able to sensitize *Bipolaris sorokiniana* and *Rhizoctonia solani* to tebuconazole and azoxystrobin, respectively (Shcherbakova et al., 2020a). 6-DMM is produced by some fungi (Endo et al., 1976; Brown et al., 1976). It belongs to the family of statins, which regulate the cholesterol content in mammals (Endo, 1985) and are used for production of some dietary products (Kim et al., 2009; Syed and Ponnusamy, 2018). It has been reported that this metabolite is also able to inhibit mycotoxin production and melanogenesis in *Aspergillius flavus* and *Fusarium* species (Dzhavakhiya et al., 2016; Mikityuk et al., 2022). Presented now, in this report, are data showing inclusion of 6-DMM with the fungicides resulting in synergistic enhancement of protective efficacy of lowered Folicur^®^ doses against a highly pathogenic wild strain of *P. nodorum*, B/8-47, in foliar treatments of wheat seedlings. In order to study the potential of 6-DMM as a sensitizing agent of resistant strains, we obtained mutants of *P. nodorum* able to grow on nutrient media containing tebuconazole or azoxystrobin at concentrations lethal to the sensitive parental *P. nodorum* strain, M-4. Once these resistant strains were obtained, we demonstrated the ability to sensitize the most tebuconazole-resistant strain *in vitro via* combining 6-DMM with Folicur^®^. Considering tebuconazole is often formulated with strobilurins to control SNB, we also determined whether 6-DMM increased sensitivity of an azoxystrobin-resistant strain to Quadris^®^ (25% azoxystrobin) by a applying a 6-DMM-fungicide mixture on tissues of detached wheat leaves.

## Materials and Methods

### Fungi

The original culture of *P. nodorum* M-4 from the State Collection of Plant Pathogenic Microorganisms (SCPPM) at the All-Russian Research Institute of Phytopathology (ARRIP) was used to produce pathogenic strains resistant to tebuconazole or azoxystrobin.

A sample of wheat kernels infected with conidia of a *P. nodorum* isolate, B-8/47, highly pathogenic to spring wheat, was supplied by the ARRIP Department of Mycology. This sample was used to prepare conidial suspensions for inoculation of wheat seedlings in greenhouse experiments.

The experimental sensitizer, 6-demethylmevinolin (6-DMM), was isolated from culture liquid of super-producer strain 18-12 of *Penicillium citrinum* developed in the Department of Molecular Biology of ARRIP (Ukraintseva et al., 2006) and maintained in our working collection.

### Fungicides and 6-Demethylmevinolin

In this study, tebuconazole and azoxystrobin, a triazole and strobilurin fungicide, respectively, were applied from commercial formulations, Folicur^®^ EC 250 (tebuconazole, 25%) and Quadris^®^ SC 250 (azoxystrobin, 25%).

To produce fungicide-resistant strains of *P. nodorum*, stock preparations of fungicides were prepared by dissolving commercial formulations in a minimum volume of dimethyl sulfoxide (DMSO), followed by dilution with ethanol. For Petri plate sensitization assays, including culturing *P. nodorum* on potato dextrose agar (PDA) as well as plant treatments, aqueous solutions of fungicidal preparations were applied. Prior to addition to PDA, fungicidal solutions were sterilized by filtration through a 0.22-µm Millipore membrane.

The candidate sensitizer, 6-DMM, was extracted from *P. citrinum* 18-12 culture liquid, whose pH was adjusted to 8.0–8.5, with acetone (1:2 v/v). The obtained extract was diluted with methanol. The supernatant of the diluted extract, containing 6-DMM in lactone configuration, was separated by centrifugation (7,500 *g*, 5 min) and concentrated by rotary evaporation. A minimal volume of ethanol was added to the concentrate and then heated at 70°C until completely dissolved. Isolated 6-DMM was purified from co-extracted compounds and quantified by HPLC (ReproSil ODS-A C18, 5 µm, 250 × 4.0 mm, Dr. Masch GmbH, Germany) using a Waters 1525 Breeze HPLC system equipped with a Waters 2487 UV detector at 234 nm. Methanol:0.1% acetic acid (80:20 v/v) was used as the mobile phase. 6-DMM lactone was converted to the water-soluble Na-salt of 6-DMM as described previously (Shcherbakova et al., 2020a) and additionally explained in **Supplementary Materials** (**Supplementary Figure 1**). The identity and purity of 6-DMM Na-salt were verified by a comparison of the obtained preparation with the corresponding commercial standard (Sigma-Aldrich, St. Louis, MO, USA) analyzed in parallel under the same conditions (**Supplementary Figure 1**). The 6-DMM Na-salt preparation was diluted with distilled water to a final 6-DMM concentration of 10% and stored for a month. On the day of each *in vitro* experiment, a portion of 6-DMM Na-salt stock preparation was mixed with ethanol to obtain a 1% solution of sensitizer, whose aliquots were pipetted aseptically into sterilized, warm melted PDA prior to inoculations by the fungus. Control *P. nodorum* colonies were grown on PDA supplemented with the same amount of ethanol as in these aliquots. Portions of the 6-DMM Na-salt stock preparation used in greenhouse experiments or detached leaf assays were diluted with sterilized distilled water prior to treatment of plants or leaves.

### Production of Fungal Mutants

#### Stimulating *P. nodorum* Sporulation on Artificial Nutrient Media

Stock cultures of the initial *P. nodorum* M-4 strain, maintained on PDA slants, were resumed by culturing for 20 days in Petri plates on Czapek Dox agar (CDA) to obtain fungal colonies. Portions of aerial mycelia from these colonies, at the log growth stage, were placed into the center of 90-mm polystyrene Petri plates containing modified CDA containing wheat leaf fragments in order to stimulate plentiful spore production by the cultured pathogen. Three days post-inoculation, sporulation was additionally stimulated by exposing plates to near-UV light (Solomon et al., 2006) for 10–14 days. To prepare spore suspensions, sterile distilled water (SDW) was pipetted onto plates, fungal conidia were gently scraped from colony surfaces, and suspensions were filtered through sterile cotton wool to remove mycelium debris. Conidial suspensions were diluted to a concentration of 5 × 10^6^ conidia/ml and used for production of fungicide-resistant mutants.

#### Random Mutagenesis and Selection of Resistant Mutant Strains

To induce and increase frequency of mutations, a short-wavelength UV-light, emitted by a Mineral Light G-80 lamp (36 W, λ 210–320 nm, UV Systems, Inc., USA), was applied as a mutagenic factor. Dose rate, exposure time, and number of irradiation cycles were previously optimized based on particular experiments analyzing survival curves of fungal conidia. Sterile conidial suspensions (10 ml) in open Petri plates placed in a laminar flow hood at the distance of 40 cm from the lamp were irradiated for 20–25 minutes at continuous gentle agitation.

Suspensions of irradiated and non-irradiated (control parental strain) conidia, at the same concentration, were diluted with SDW, evenly spread (0.1 ml of suspension) onto the surface of 90-mm CDA Petri plates already supplemented with Folicur^®^ or Quadris^®^ at concentrations ranging from 0.1 to 1.0 ppm. Conidial dilutions, used for CDA inoculation, were adjusted so that 50 to 100 colonies appeared per plate. After inoculation, plates were incubated in the dark at 22°C for 7–10 days to reveal mutant lines able to grow in the presence of the fungicides better than parental strain M-4.

Irradiated colonies with diameters twice that of non-irradiated (controls) on media with 1.0 ppm of fungicides were selected to obtain a second generation of mutants able to grow on CDA supplemented with fungicidal doses of 2.5 to 12.5 ppm, at intervals of 2.5 ppm.

To confirm irradiated fungicide-resistant mutants developed colonies on media with fungicide concentrations at sub-lethal levels (levels at which control strains could develop) and maintain acquired resistance, selected mutants were consecutively cultured, first on fungicide-free CDA and then on fungicide-free PDA. Mutants were then tested for resistance, again, by re-culturing on fungicide-containing agar. The two most resistant strains for each fungicide tested were selected and maintained on PDA supplemented with 1.0 ppm of the respective fungicide, until use in sensitization experiments. The tebuconazole-resistant strain was designated “m8-4” and that resistant to azoxystrobin was designated “kd-18”.

The parental strain M-4 and resistant mutants selected for *in vitro* and *in vivo* testing were sub-cultured every 3 weeks on PDA and fungicide-containing PDA, respectively.

### *In Vitro* Evaluation of 6-DMM Sensitizing Effect Towards *P. nodorum* Resistant to Tebuconazole

Methods for sensitization experiments involving checkerboard assays and multifold double-dilution tests were previously reported (Canton et al., 2005; Orhan et al., 2005; Campbell et al., 2012). The Limpell method (Richer, 1987) used for the determination of the synergistic effect involving 6-DMM/fungicide combinations has also been reported previously (Shcherbakova et al., 2020a). Briefly, 6-DMM sensitizing activity towards the tebuconazole-resistant strain (m8-4) was evaluated by cultivation on PDA containing Folicur^®^ at sub-fungicidal concentrations combined with 6-DMM at non-fungicidal or marginally fungicidal doses. In parallel, cultures were grown on PDA supplemented with fungicide alone, or 6-DMM alone, at respective concentrations to those used in co-application treatments. To select sub-fungicidal concentrations suppressing growth of a non-resistant pathogen by less than 50%, preliminary experiments were carried out. The range of 6-DMM concentrations presumably suitable for sensitization of *P. nodorum* were calculated previously (Shcherbakova et al., 2020a). In addition, minimal inhibitory concentrations (MICs) of 6-DMM, Folicur^®^, and Quadris^®^ preventing a visible colony growth of wild M-4 and B-8/47 strains as well as 6-DMM MICs for the two most resistant mutants (m8-4 and kd-18), and MICs of Folicur^®^ and Quadris^®^ for m8-4 and kd-18, respectively, were determined according to Espinel-Ingro et al. (2012) (**Supplementary Table 1**). Control cultures were grown under the same conditions on non-supplemented PDA. Growth inhibition percent was calculated by the level of diminished average colony diameter, compared to controls, based on measuring diameters of each colony in two perpendicular directions (six replications per treatment for each strain).

### Assessment of the Sensitization Effect Using Treatments of Wheat Seedlings or Isolated Wheat Leaves

#### Bioassay on Detached Wheat Leaves Treated With 6-DMM Combined With Quadris^®^

To reveal a possible synergy between 6-DMM and azoxystrobin towards suppression of disease symptoms resulting from inhibition of conidial germination on infected leaves, and to determine *in vivo* sensitization of azoxystrobin-resistant kd-18, a detached leaf assay was performed as previously described in detail (Shagdarova et al., 2018; Shcherbakova et al., 2021; Voinova et al., 2021). In summary, Quadris^®^ and 6-DMM were co-applied to fragments of detached wheat leaves placed in Petri plates containing water agar. These fragments were cut from fully expanded first leaves of wheat seedlings (cv. Khakasskaya) grown to the two leaf stages. Top portions of leaf fragments (10 per each combined or individual treatment) were inoculated with 10-µl droplets of fungal conidia suspended (10^6^ spore/ml) in the fungicide (2.5, 5.0, or 7.5 ppm), 6-DMM solutions (10 ppm), or in mixtures of the two. Bottom portions of the same leaf fragments were treated with 10-µl droplets of the conidial suspension in SDW (control). Average disease index was determined based on severity of disease symptoms observed 5 days post-inoculation, using a five-score rating scale (Shagdarova et al., 2018).

The detached wheat-leaf assay with conidial suspensions applied in the middle of leaf fragments (no 6-DMM or Folicur^®^ treatments) was also employed to confirm the ability of tebuconazole-resistant mutant m8-4 to infect wheat.

#### Greenhouse Experiments on Treatments of Wheat Seedlings with 6-DMM Combined With Folicur^®^

Seeds of spring wheat seedlings (cv. Khakasskaya) were surface-disinfected by immersion in 0.5% KМnO_4_ for 5 min, washed, germinated on damp sterilized paper, and sown in pots placed in a greenhouse box. Seedlings (100 per treatment, 25 seedlings per pot) were grown at ambient temperature to Z12–Z13 stages, according to the Zadoks growth scale (Zadoks et al., 1974). Seedlings were then treated with Folicur^®^, at sub-fungicidal dose rates, 6-DMM, or in combination with the two components. Seedlings were inoculated with pathogen conidia either simultaneously with singular or combined treatments (preventive treatments) or after treatments (curative treatments). Inoculation suspensions were prepared by rinsing B-8/47 conidia from infected wheat grain. Pots with inoculated or treated and inoculated seedlings were placed into an inoculation chamber filled with water mist and incubated at 18°C for 24 h to promote infection. For preventive treatments, conidial suspensions were supplemented with Folicur^®^ alone, 6-DMM alone, or Folicur^®^ combined with 6-DMM to experimental concentrations. In post-inoculation (curative) treatments, seedlings were sprayed with *P. nodorum* conidia a day before spraying with aqueous solutions of Folicur^®^ alone, 6-DMM alone, or Folicur^®^ combined with 6-DMM. Folicur^®^ was applied at two separate dose rates equivalent to 10% and 20% of recommended rates for field treatments of wheat; 6-DMM was applied at 10 ppm, and a final concentration of conidial suspension was adjusted to 10^6^ conidia/ml (**Table 1**). Treatments and inoculation of seedlings were performed using a portable ultra-low volume plant sprayer. The volume of working fluid (30 ml per 100 plants) was calculated based upon volume rate recommended for field treatments.

**Table 1:** List of preventive or curative seedling treatments that were used in greenhouse experiments.

#	Treatments
1	Control (neither Folicur^®^ nor 6-DMM)
2	6-DMM, 10 ppm
3	Folicur^®^ EC 250, 320 ppm (10% of RDR*)
4	Folicur^®^ EC 250, 640 ppm (20% of RDR)
5	Folicur^®^ EC 250, 320 ppm + 6-DMM, 10 ррm
6	Folicur^®^ EC 250, 640 ppm + 6-DMM, 10 ррm
7	Folicur^®^ EC 250, 3200 ppm (recommended dose rate)

* RDR—recommended dose rate.

Disease severity on treated and non-treated (control) inoculated plants was scored according to the international James’ scale (James, 1971) 14–15 days after inoculation as recommended for recording of early disease symptoms (Sanin and Sanina, 2013).

Protection efficacy (PE,%) of treatments was evaluated by percent suppression of disease compared to controls. Synergistic interaction between 6-DMM and the fungicide was determined by comparing PE,% values obtained in combined treatments (PEr,%) with the level of an expected additive effect of individual treatments (PEe,%) calculated using the Limpell formula, as described previously (Shcherbakova et al., 2021).

### Statistics

Results were analyzed using STATISTICA 6.1 software (StatSoft Inc. Tulsa, OK, USA) to calculate mean values, standard deviations, standard errors, and approximation confidence coefficients (*R* ^2^). Significant differences between means of treatments and controls were set at *p* ≤ 0.05 (*t*-test for independent variables). Experiments were performed in triplicate.

## Results

### Characterization of *P. nodorum* Strains Resistant to Tebuconazole or Azoxystrobin

**Figure 1:**
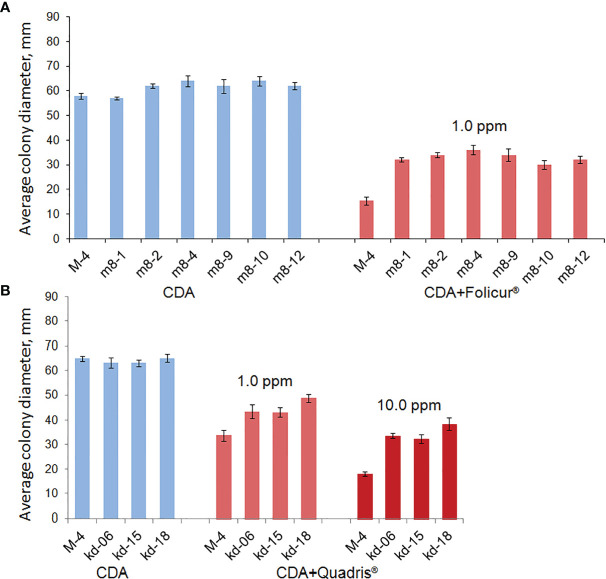
Rates of radial growth of *P. nodorum* wild strain (M-4) and mutants (m8-2, m8-4, m8-9, m8-10, and m8-12) resistant to Folicur^®^ or Quadris^®^ on Czapek Dox agar (CDA) and CDA supplemented with fungicides. Mean colony diameters were calculated after measuring the diameters of each 7-day colony in two perpendicular directions (3 colonies per treatment in each of the thrice-repeated experiments). Columns indicate the mean of three experiments, six replications per strain. Y-bars show SD.

After UV irradiation of conidial suspensions of the parental *P. nodorum* M-4 strain and exposure to fungicides in concentrations ranging from 0.1 to 1.0 µg/ml, we selected 25 and 19 candidate mutant lines possessing a lowered sensitivity to tebuconazole and azoxystrobin, respectively, compared to strain M-4. Among the lines selected, three colonies from CDA with 1.0 ppm of Quadris^®^, and six colonies grown on CDA supplemented with Folicur^®^ at 1.0 ppm had diameters approximately twice that of non-irradiated control M-4 colonies, grown under the same conditions (**Figure 1A**).

**Figure 2:**
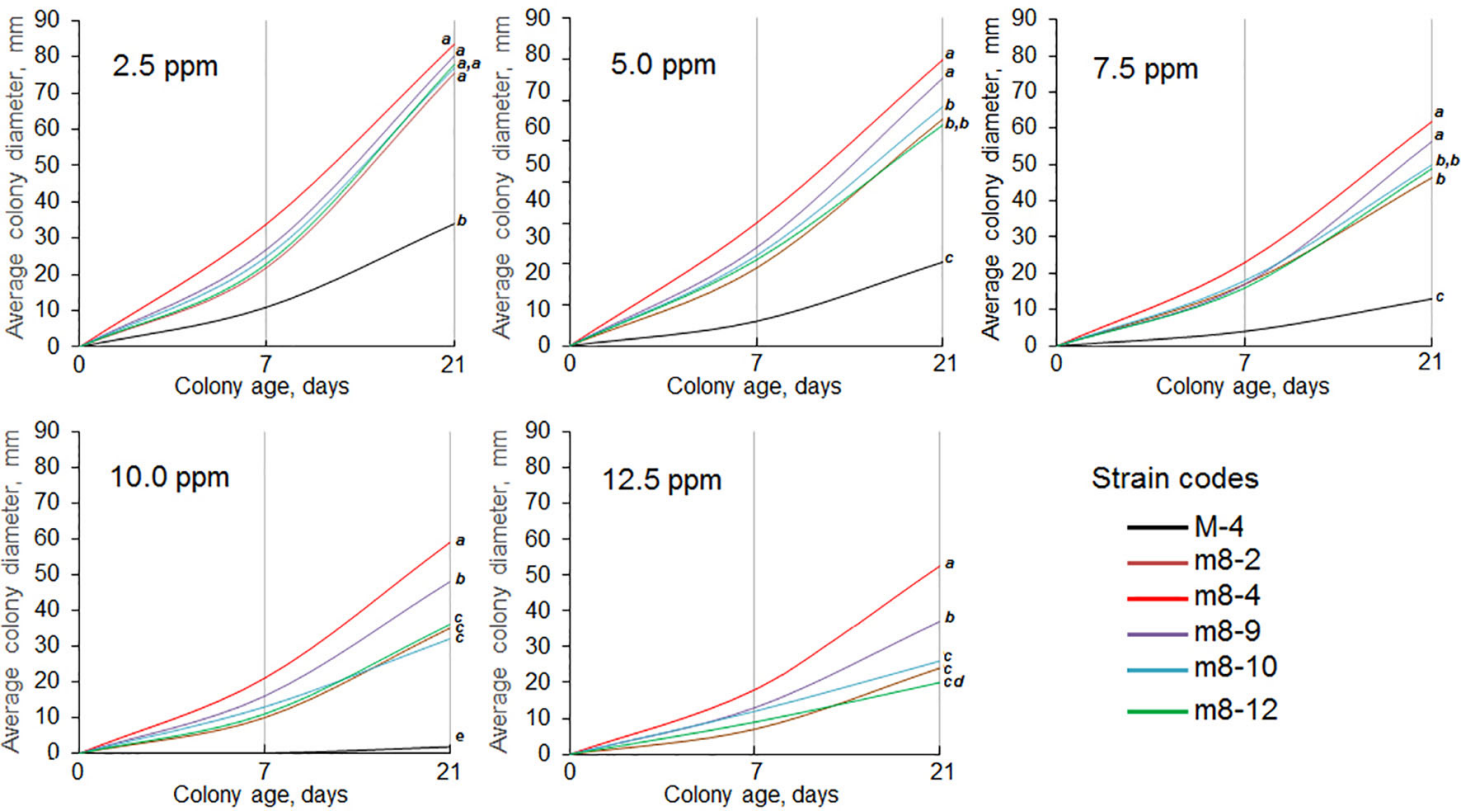
Dynamics of radial growth of five *P. nodorum* strains resistant to tebuconazole cultured on CDA containing Folicur^®^ at concentrations ranging from 2.5 to 12.5 ppm. Strain M-4 is the parental, wild strain. Differences between average colony diameters marked with the same bold italic letters, at the right margin of the graphs, show insignificant (*p* > 0.05) differences by termination of cultivation. For additional explanation, see **Figure 1**.

**Figure 3:**
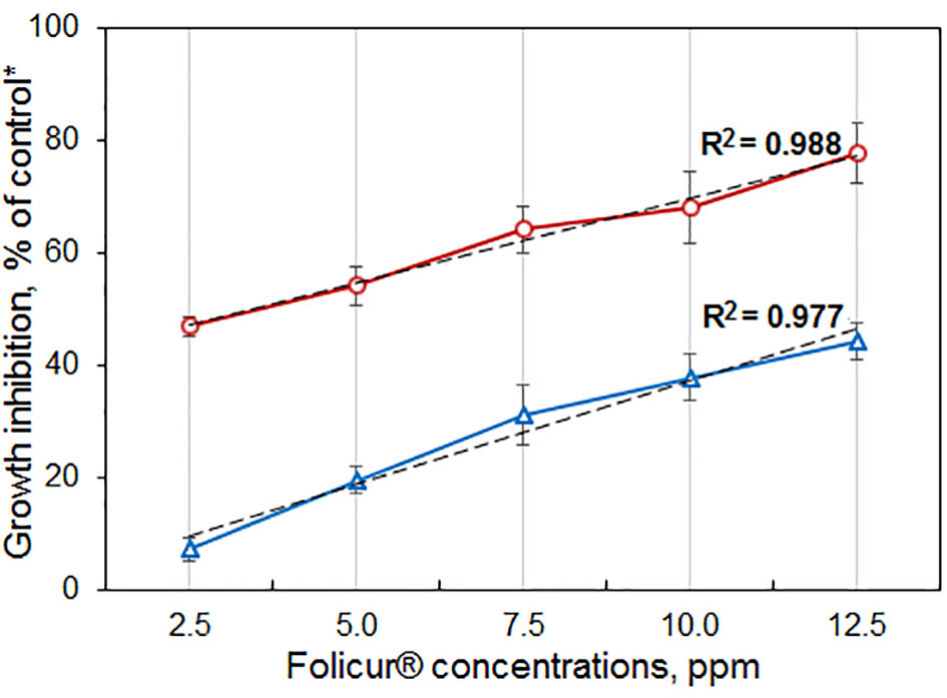
Dependence of *P. nodorum* m8-4 growth inhibition on concentrations of Folicur^®^ incorporated into potato dextrose agar (PDA). *Colonies grown on Folicur^®^-free PDA served as control. Dotted lines indicate lines corresponding to linear function. *R* ^2^ values and Y-bars show confidence coefficients and SD, respectively. Red and blue lines are percent growth inhibition after 7 and 21 days, respectively.

The six mutant colonies revealed on the Folicur^®^ containing CDA (denoted as strains m8-1, m8-2, m8-4, m8-9, m8-10, and m8-12) developed normally when cultured on CDA supplemented with Folicur*^®^* up to a concentration of 7.5 ppm, a dose close to lethal for M-4. However, the m8-1 strain did not retain resistance to tebuconazole after two passages through Folicur*^®^*-free CDA and PDA. Since none of the other strains reverted to tebuconazole sensitivity, they were selected for further research as tebuconazole-resistant forms of the pathogen (**Figure 2**). Two strains (m8-4 and m8-9) produced the largest colonies when cultured on media containing Folicur^®^ at 10.0 ppm, lethal for non-resistant wild M-4 (**Figure 2**). Strain m8-4 was the most resistant to tebuconazole, exhibiting colony growth much faster in the presence of Folicur^®^ than the other four strains, even in the presence of Folicur^®^ at a concentration of 12.5 ppm (**Figure 2**). In addition, this strain was more responsive to changes of fungicide concentration compared to the other resistant strains. The dependence of m8-4 radial growth was characterized by almost a linear negative correlation to increased Folicur^®^ concentrations (from 1.0 to 12.5 ppm) at both beginning (7th day), and the end (21st day) of observations (**Figure 3**).

The parental M-4 strain was initially much less sensitive to azoxystrobin than to tebuconazole (**Supplementary Table 1**). However, we were able to develop strains with elevated resistance after irradiation, resulting in the three aforementioned mutant colonies. The colonies of these strains grew twice as fast on media containing 1.0–10.0 ppm of Quadris^®^ compared to M-4 (**Figure 1B**). However, two of these strains lost their resistance to Quadris^®^ upon passaging through fungicide-free media. The third strain (denoted as kd-18) retained its resistance to azoxystrobin after passaging. This strain, when cultured on gradually increasing concentrations of Quadris^®^ (25–100 ppm), retained its resistance until the end of the 21-day experiment. Under the same conditions, mycelia growth and conidia germination of M4 was severely to completely inhibited (**Table 2**).

**Table 2:** Sensitivity of the most resistant *P. nodorum* mutant strain (kd-18) to azoxystrobin-based Quadris^®^ SC 250 compared to the parental strain (M-4).

Concentration of Quadris^®^, SC 250, ppm	Growth inhibition, % relative to control* (mean ± SD)			
	At day 7		At day 21	
	M-4	kd-18	M-4	kd-18
25.0	84.5 ± 3.5	53.0 ± 5.6	60.0 ± 4.2	23.6 ± 3.2
50.0	89.7 ± 3.3	65.6 ± 3.4	91.8 ± 1.9	28.5 ± 1.6
75.0	99.1 ± 0.7	75.0 ± 7.5	96.2 ± 3.8	42.7 ± 4.4
100.0	100.0 ± 0.0	79.6 ± 6.3	100.0 ± 0.0	49.3 ± 3.0

*Control colonies were cultured on media free of Quadris^®^.

**Figure 4:**
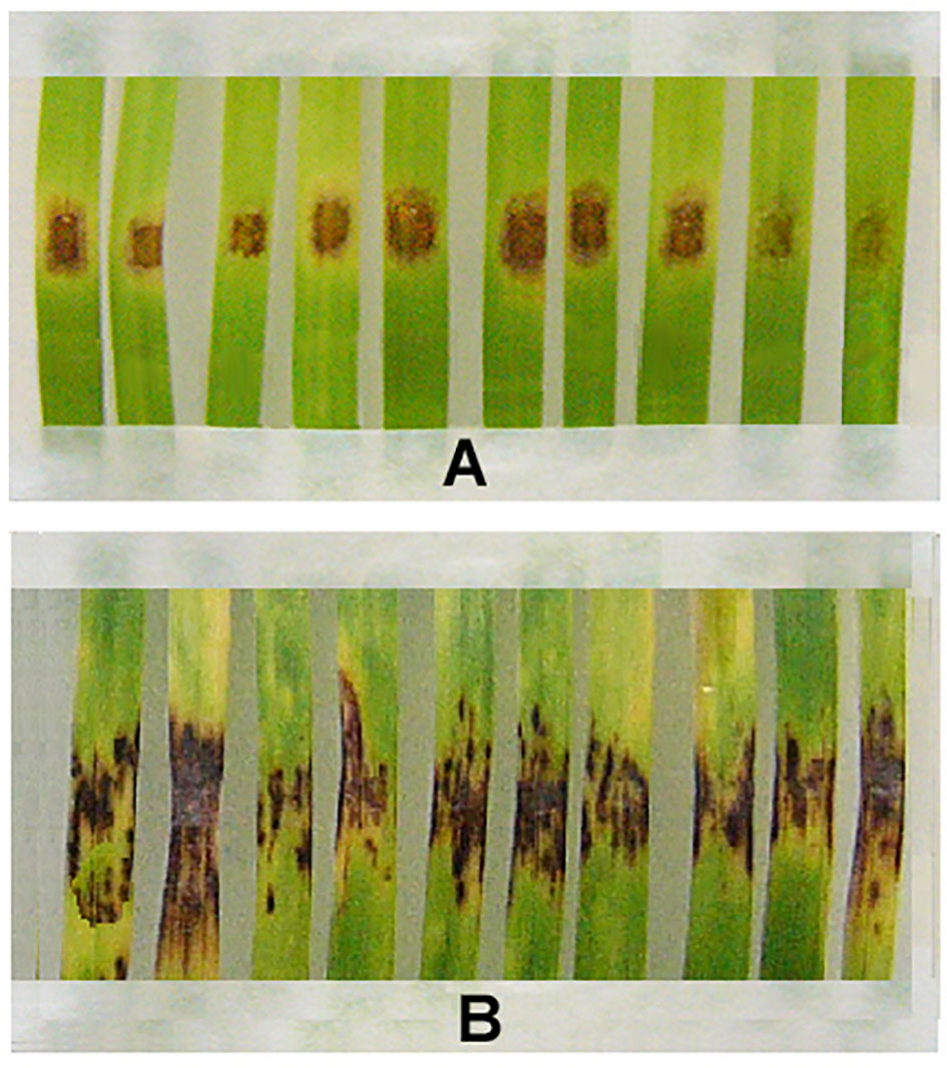
Disease symptoms on fragments of detached wheat leaves inoculated with the Folicur^®^-resistant strain, m8-4 **(A)**, or the Folicur^®^-susceptible, wild strain of *P. nodorum*, M-4 **(B)**. Conidial suspensions applied at a final concentration of 10^6^/ml.

Although resistant strains produced fewer spores than wild parental M-4, bioassays on detached wheat leaves using conidia collected from m8-4 and kd-18 colonies showed that these strains were able to infect leaf fragments. However, disease symptoms of m8-4 infection were reduced compared to infection by the wild strain (**Figure 4**). Like the parental wild strain, mutant kd-18 was able to infect wheat. The disease index (DI) on control detached leaves inoculated with kd-18 conidia was the same as after inoculation of control leaves with conidia of the parental strain (**Table 3**).

**Table 3:** Influence of Quadris^®^ SC 250, 6-DMM or their combinations on the disease severity on detached wheat leaves infected with *P. nodorum* wild strain (M-4) or azoxystrobin-resistant mutant (kd-18).

Treatments and concentrations, ppm	Average disease index* (mean ± SE)		Percent of disease suppression (Er/Ee**)	
	M-4	kd-18	M-4	kd-18
Control (neither 6-DMM nor fungicide)	3.9 ± 0.09	3.5 ± 0.38	−	−
6-DMM, 10.0	2.6 ± 0.31	2.7 ± 0.11	33.3	22.9
Quadris ^®^, 0.5	2.4 ± 0.25	3.0 ± 0.00	38.4	14.3
Quadris ^®^, 7.5	1.8 ± 0.03	2.4 ± 0.09	53.8	31.4
Quadris ^®^, 0.5 + 6-DММ, 10	0.9 ± 0.04	2.2 ± 0.15	76.2^a^/*58.6^b^*	37.1/33.9***
Quadris ^®^, 7.5 + 6-DММ, 10	0.6 ± 0.05	1.2 ± 0.04	84.4^c^/*69.2^d^*	65.7^a^/*47.1^b^*

*Score scale: 1—small dark necroses (1–2 mm in diameter); 2—dark brown clearly bordered growing spots without chlorosis; 3—large light brown or brown spots edged by chlorosis; 4—brown rapidly growing spots without clear borders, pycnidia occasionally formed.

**Numbers before the slash (/) and italicized numbers following slash indicate Er and Ee values (a calculated additive effect), respectively. The difference between these values marked with different letters within each column are significant at p ≤ 0.05.

***The case of additive effect (difference between Er and Ee is insignificant at p ≤ 0.05).

Based on the above observations on sensitivity of *P. nodorum* mutants to fungicides, as induced by random mutagenesis, we selected resistant strains m8-4 and kd-18 for further experiments on *in vitro* and *in vivo* sensitization to overcome resistance to tebuconazole and azoxystrobin, respectively.

### *In Vitro* Sensitization of a *P. nodorum* Resistant to Tebuconazole

**Figure 5:**
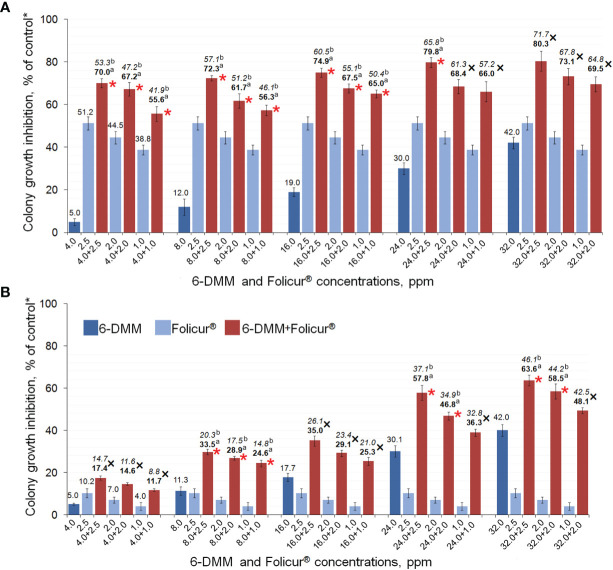
The enhanced inhibitory effect of 6-DMM combined with Folicur^®^ EC 250 on *in vitro* growth of wild M-4 **(A)** and resistant m8-4 **(B)** strains of *P. nodorum*. *Control colonies were cultured on potato dextrose agar free of 6-DMM and fungicide. Each histogram column represents the mean of three experiments. Y-bars indicate SD. The numbers in bold or regular above the columns indicate Er values, while italic numbers show Ee values related to the same fungicide-6-DMM concentration combination. Er indicates inhibition (%) of the colony growth when fungicide and 6-DMM were co-applied; Ee is the estimated inhibition (%) of colony growth if there is an additive effect of fungicide and 6-DMM. Where Er > Ee at *p* ≤ 0.05, a synergistic interaction between fungicide and sensitizer is confirmed. The 6-DMM-Folicur^®^ combinations resulting in a synergistic effect (Er > Ee at *p* ≤ 0.05) towards M-4 **(A)** and m8-4 **(B)** are designated with red asterisks placed near columns. Different letters **(a, b)** indicate significant difference between Er and Ee (95% CI). In five **(A)** and eight **(B)** other cases marked with a black “**×**”, 6-DMM-Folicur^®^ interactions were additive (Er > Ee at *p* > 0.05). Ee values are calculated by the formula Ee = (X + Y) – XY/100 (Richer, 1987), where X and Y represent percent growth inhibition by each of the components alone.

Our experiments involving growing of M-4 or m8-4 on 6-DMM-containing PDA showed that the wild and tebuconazole-resistant strains did not differ in their sensitivity to 6-DMM. The 6-DMM MIC amounted to 5.01 mg/ml and 4.90 mg/ml for M-4 and m8-4, respectively. These values were one or two orders of magnitude lower than MICs of Folicur^®^, which amounted to 12.5 µg/ml and 112.5 µg/ml for the sensitive strain M-4 and resistant strain m8-4, respectively (**Supplementary Table 1**). Consequently, in our experiments, 6-DMM showed low fungitoxicity towards *P. nodorum* that is typical for many other known chemosensitizers, which generally have low or mild antimicrobial activity (Campbell et al., 2012). At the same time, both strains responded to the sensitizing impact of 6-DMM. Its co-applications with Folicur^®^ at different concentration combinations significantly exacerbated growth inhibition towards both strains compared to the inhibitory effect of the fungicide alone (**Figures 5A, B**). Among the 15 concentration combinations of the fungicide with the sensitizer, tested by the checkerboard assay in our experiments, 10 combinations used for M-4 growth inhibition (**Figure 5A**) and seven applied against m8-4 (**Figure 5B**) resulted in a synergistic increase of Folicur^®^ effect (Er > Ee at *p* ≤ 0.05). Percentage of radial growth inhibition obtained with combined treatments (Er) exceeded the calculated inhibition percentage expected for an additive effect (Ee) when M-4 was cultured on any of the Folicur^®^ concentrations (from 1.0 to 2.5 ppm) in combinations with 6-DMM, at doses from 4 to 24 ppm (**Figure 5A**). Synergistic augmentation of the resistant m8-4 sensitivity to tebuconazole (Er > Ee at *p* ≤ 0.05) was observed if the fungicide (1.0–2.5 ppm) was combined with 8 ppm of 6-DMM, as well as at the four 6-DMM: Folicur ratios, viz., at 24:2.5 ppm, 24:2.0 ppm; 32:2.5 ppm, and 23:2.0 ppm (**Figure 5B**). In other cases, the effect of combined treatments was mainly additive (Er > Ee at *p* > 0.05). A number of combinations of 6-DMM at 8 ppm + Folicur^®^ in the range from 1.0 to 2.5 ppm produced synergistic growth inhibition of both the sensitive M-4, and the resistant strain m8-4 (**Figures 5A, B**). In addition, a synergistic combination of 6-DMM with the fungicide at 24.0 ppm and 2.5 ppm, respectively, was also found for sensitive M-4 (**Figure 5A**) and resistant m8-4 (**Figure 5B**). The maximum inhibitory effect from combinations belonging to overlapping synergistic ranges indicated amounted to 72.3 or 79.8% for the sensitive strain (**Figure 5A**) and 33.5 or 57.8% for the resistant m8-4 (**Figure 5B**).

### Sensitization of *P. nodorum* to Azoxystrobin Using Plant Tissue Treatments With Quadris^®^ Combined With 6-DMM

The ability of 6-DMM to enhance fungal sensitivity to azoxystrobin was demonstrated using bioassays with detached wheat leaves. Wheat leaf fragments were inoculated with conidia of *P. nodorum* parental wild strain, M-4, or the isolated resistant mutant, kd-18, suspended in either aqueous solutions of 6-DMM combined with Quadris^®^ or in solutions of each individual component at the same concentrations used in combined suspensions (**Table 3**). Combined treatments using leaf tissue assays showed a 1.5- to 2.0-fold reduction of disease indices from infections by either M-4 or kd-18, compared to applications of Quadris, alone. Reduction in disease symptoms after co-applications of Quadris^®^ (at 5.0 or 7.5 ppm) and 6-DMM (at 10 ppm) on detached leaves inoculated with the wild strain resulted from a synergistic interaction between the fungicide and the sensitizer as confirmed by a significantly greater (*p* ≤ 0.05) Er than Ee. In addition, combinations of Quadris^®^ 7.5 ppm and 6-DММ 10 ppm also showed reduced disease symptoms from inoculations with the Quadris^®^-resistant strain, kd-18, as a result of a synergistic interaction between the fungicide and the sensitizer.

### Enhancement of the Fungicidal Tebuconazole Effect in Treatments of Wheat Seedlings With 6-DMM Combined With Folicur^®^

**Figure 6:**
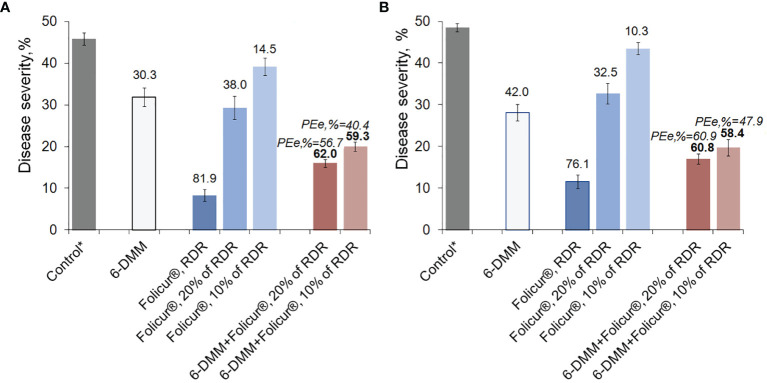
Levels of disease development on wheat plants (cv. Khakasskaya) infected by a highly pathogenic strain (B-8/47) of *P. nodorum* after plants were treated with Folicur^®^ EC 250, the sensitizer (6-DMM), or 6-DMM combined with Folicur^®^. **(A)** Preventive treatments (plants treated with fungicide simultaneously with inoculation). **(B)** Curative treatments (plants sprayed with fungicide one day after inoculation). *Control—disease severity on non-treated plants after inoculation with *P. nodorum* B8/47 conidia (maximum score of leaf damage according to the James scale, 1971 is 50%). RDR = recommended dose rate. The numbers in bold or regular above the columns indicate PEr,%; values of Y-bars indicate SE with a 95% confidence interval (*n* = 100, *p* = 0.05). For additional explanation, see **Figure 5** and section “*Materials and Methods*,” point 3.4.

Greenhouse experiments on treatments of wheat seedlings confirmed the ability of 6-DMM to enhance the protective effect of Folicur^®^ against *P. nodorum*, not only *in vitro*, but also when plants were artificially infected with this causative agent (**Figure 6**). In these experiments, 6-DMM was applied at a concentration 10 ppm, which was five times lower than its ED_50_ for the highly pathogenic *P. nodorum* strain, B-8/47, used for plant inoculations (Shcherbakova et al., 2020a). Also, this dose was in the concentration range providing only 10%–20% growth inhibition of M-4, another wild pathogenic strain, shown in the experiments outlined above, to enhance its sensitivity to tebuconazole, *in vitro* (**Figure 5A**).

The disease suppression activity of 6-DMM, alone, on plants was found to be higher than the growth inhibitory effect on the pathogen revealed *in vitro*. At the used concentration of 10 ppm, protection efficacy of 6-DMM averaged 30.0 ± 2.2% and 40.0 ± 2.0% in preventive treatments (when applied simultaneously with inoculations) and curative (inoculation by conidia one day after treatments), respectively.

Depending on mode of treatment, spraying seedlings with Folicur^®^ alone at 10% or 20% of recommended dose rate (RDR) resulted in, at best, only a 1.2- to 1.5-fold decrease of disease severity, compared to non-treated controls. Conversely, both methods of treatment (preventative or curative) with 20% or 10% Folicur^®^ combined with 6-DMM at 10 ppm significantly improved the protective effect of the fungicide at doses lower than RDR. In preventive treatments, disease severity decreased by twofold compared to controls. In curative treatments, co-application of the fungicide and the sensitizer resulted in almost threefold reduction in disease severity.

A synergistic effect was observed in both preventive and post-inoculation treatments, when the sensitizer was co-applied with a 10% RDR of fungicide. At this combination rate, disease reduction was significantly higher than the maximum level of disease suppression from individual application of Folicur^®^ at 10% or 20% RDR, and exceeded what would have been expected for an additive effect. PEr,% values significantly exceeded (*p* ≤ 0.05) PEe,% for all combinations of the fungicide and the sensitizer in all experiments, and ranged from 10.5% to 19% greater in curative and preventive treatments, respectively (**Figure 6**). Moreover, the level of protective effect (PEr,%) in preventive treatments using combinations of 6-DMM at 10 ppm and Folicur^®^ at 320 ppm (10% of RDR) was significantly higher than after application of fungicide alone at twice the concentration, 640 ppm (20% of RDR) (**Figure 6A**). Similar results were obtained when separate and combined treatments with the sensitizer and the tebuconazole formulation, both at aforementioned doses, were carried out one day after inoculation of plants (**Figure 6B**). Improvement of plant protection using mixtures of sensitizer with Folicur^®^ at 20% RDR resulted from an additive effect (**Figure 6B**).

## Discussion

To combat plant pathogenic fungi causing diseases of agricultural crops, a variety of plant protection technologies, alternative to fungicidal treatments, is currently available (Berg, 2009; Rosentrater and Evers, 2018; Silva et al., 2018). To date, application of fungicides remains a principal strategy for obtaining high yields and high-quality products for the agricultural industry, worldwide. However, crop protection based on modern xenobiotic fungicides has several serious drawbacks. A fundamental issue with wide-scale application of fungicides is associated with their ecological and human health risks (Carvalho, 2017). Another problem of extreme concern is the development of resistance to chemical fungicides by plant pathogens, especially to compounds whose mode of action targets a singular metabolic or structural component in the pathogen. Such fungicides include triazoles and strobilurins, inhibitors of sterol biosynthesis and mitochondrial respiration, respectively (Oliver, 2014; Lucas et al., 2015).

The main problem involved with resistance to fungicides is the treadmill of an increasing requirement to augment application dosage and frequency to achieve effective control as pathogens develop more and more resistance. This need for increased dosage and frequency results in greater environmental contamination expense to the grower and development of resistant strains of pathogens. Hence, from these standpoints, searching for ways to improve fungicidal toxicity and overcoming pathogen resistance is paramount.

Chemosensitization of plant pathogenic fungi to fungicides using environment-friendly, natural compounds (sensitizers) is a promising strategy to overcome this problem (Campbell et al., 2012). A number of studies clearly suggest that the chemosensitization-based approach has a powerful potential in sustainable agriculture. This approach may provide proper control of fungi while reducing effective doses of triazoles, strobilurin, and some other fungicides required for fungal pathogen control (Kim et al., 2007; Dzhavakhiya et al., 2012; Shcherbakova, 2019). Indeed, co-application of commercial strobilurin and triazole formulations with sensitizers, which impair pathogen metabolism or interfere with cell wall structures, result in enhanced pathogen sensitivity to antifungal agents and synergistic augmentation of their inhibitory impact on mycelial growth and conidia germination *in vitro* (Yen and Chang, 2008; Kim et al., 2010; Dzhavakhiya et al., 2012; Odintsova et al., 2020; Shcherbakova et al, 2020a). Moreover, due to increased fungicidal effect, lowered dose rates of fungicide, when applied in combination with sensitizers, effectively suppress the development of various pathogens in plant tissue assays (Shcherbakova et al, 2020a), as well as in seed and foliar treatments, under controlled (Shcherbakova et al, 2021) and field (Kim et al., 2017) conditions. In addition, some chemosensitizing agents that are natural products of plant origin (viz., thymol and 2,3-dihydroxybenzaldehyde) have been reported to be a tool to enhance the sensitivity of a strain of *P. nodorum* resistant to difenoconazole (Kartashov et al., 2019) and to prevent tolerance of *Aspergillus fumigatus* to some antimycotics (Kim et al., 2008).

Previously, we investigated the ability of 6-DMM, a microbial inhibitor of HMG-CoA reductase, to suppress growth and spore germination of several plant pathogenic fungi *in vitro*, including *P. nodorum* (Shcherbakova et al., 2020a). We found that 6-DMM displayed mild antimycotic activity, typical for other known pathogen-sensitizing agents (Campbell et al., 2012). Ranges of minimally toxic and sub-toxic 6-DMM concentrations suitable for *in vitro* sensitization of *P. nodorum* and other fungi were determined experimentally and using probit analysis (Shcherbakova et al., 2020a). In the research reported here, we used concentrations of 6-DMM, within ranges determined by these prior experiments, to increase the sensitivity of susceptible and resistant *P. nodorum* strains to tebuconazole- or azoxystrobin-based agricultural fungicides, widely applied to control SNB in Russia and other countries.

The possibility to enhance tebuconazole effect was demonstrated towards two wild *P. nodorum* strains. One of them, M-4, was an SCPPM accession characterized as a tebuconazole-sensitive strain; therefore, it was used to obtain tebuconazole-resistant mutants and to improve the *in vitro* growth inhibitory effect of Folicur^®^. Another wild strain, B/8-47, representing a highly pathogenic strain for spring wheat isolate, was widespread in the majority of pathogen populations of the Moscow region. Therefore, B/8-47 conidia collected in fields and stored on infected grain were used for plant inoculations in greenhouse experiments involving sensitization of *P. nodorum* to Folicur^®^ using 6-DMM. Our previous experiments showed that B/8-47 was less sensitive (MIC = 32.0 µg/ml) to Folicur^®^ than M-4 (MIC = 12.5 µg/ml), but much more sensitive compared to the resistant mutant m8-4 with MIC 112.5 µg/ml (**Supplementary Table 1**).

We found that several concentration combinations synergistically improved the growth inhibitory effect of Folicur^®^ against a wild parental isolate, M-4, and the most tebuconazole-resistant M-4 mutant (m8-4), obtained by mutagenesis, *in vitro*. These results confirm our previous data (Shcherbakova et al., 2020a) showing the sensitizing activity of 6-DMM towards another wheat pathogen *B. sorokiniana* to Folicur^®^, and a potato pathogen *R. solani* to Quadris^®^. Importantly, in sensitization experiments, there was an overlapping range of synergistic combinations of sensitizers and fungicides effective against both the sensitive M-4 and the resistant strain m8-4 (**Figure 5**). As mentioned above, such synergistic interactions occurred if 6-DMM was applied at the marginally toxic concentration (8 ppm), combined with Folicur^®^ concentrations increasing from 1.0 to 2.5 ppm. In addition, if the pathogen was exposed to Folicur^®^ at 2.5 ppm combined with 6-DMM at 24 ppm, a high level of fungitoxicity was also observed towards M-4 and resistant m8-4. These findings suggest that 6-DMM may be considered as a promising sensitizing agent for the control of *P. nodorum* in populations containing strains of the pathogen resistant to Folicur^®^. Moreover, based on our findings, it is possible to select sensitizer and Folicur^®^ concentrations that, when co-applied, will be effective not only against strains sensitive to tebuconazole, but also against resistant strains.

Of note is the sensitization shown *in vitro* by Petri plate assays that cannot always be reproduced by *in vivo* tests on plant material. Considering this fact, we examined the ability of 6-DMM plus Folicur^®^ EC 250 treatments to enhance the protective efficacy of tebuconazole on seedlings inoculated with a highly pathogenic wild strain B/8-47 in greenhouse experiments. Using foliar treatments of wheat seedlings simultaneously with inoculation by *P. nodorum* or treating a day after inoculation, we confirmed 6-DMM-triggered sensitizing activity towards this highly pathogenic strain, B/8-47. The antifungal activity of 6-DMM, alone, applied on plants was higher than observed *in vitro*. Thus, there was greater activity by 6-DMM to synergistically augment antifungal interactions of 6-DMM/Folicur^®^ combinations, consequently improving the protective action of tebuconazole on plants. Moreover, this synergistic augmentation of Folicur^®^ antifungal activity towards *P. nodorum* B-8/47 by the sensitizer resulted in an effective dosage rate of the fungicide 10 times lower than RDR. In addition, PEr values (observed % protective efficacy) obtained in preventive and curative treatments of 6-DMM combined with the fungicide used at 10% and 20% RDR were similar in reducing fungal growth (**Figure 6**). This similarity suggests that the co-application of the compounds would be effective even after the pathogen had already infected the plant. Such curative (post-infection) treatments are usually the case under real field conditions. Thus, improved activity of 6-DMM/Folicur^®^ combinations is of interest from a practical standpoint.

The putative mode of action of the sensitizer resulting in synergistic attenuation of pathogens is of consequence. Using some natural compounds, it is possible to specifically target metabolic pathways of plant pathogenic fungi different from those targeted by modern agricultural fungicides. The use of this strategy to enhance plant protective efficacy and reduce the risk of development of resistance to fungicides of target fungi is our key endeavor. In this study, we propose that the sensitizer/fungicide synergy we observed resulted from the fact that 6-DMM targets a metabolic pathway in fungi distinct from that targeted by tebuconazole or azoxystrobun. Tebuconazole, like other triazoles, inhibits 14-alpha-demethylase, an enzyme converting 24-methylene-24,25-dihydrolanosterol to ergosterol, a major structural component of cell membranes of fungi, late in the stage of ergosterol biosynthesis (Klix et al., 2007; FRAC, 2021). Alternatively, 6-DMM blocks HMG-CoA reductase, an enzyme catalyzing an early stage of sterol biosynthesis in the mevalonate pathway. 6-DMM also effectively sensitizes *P. nodorum* to a storbilurin fungicide blocking ubiquinone oxidase, azoxystrobin, whose antifungal effect, unlike triazoles, is not associated with sterol biosynthesis (Mueller et al., 2013; FRAC, 2021). Since 6-DMM targets other sites of fungal metabolism than those by tebuconazoles or strobilurins, when used in combination, it is more difficult for the pathogen to overcome activity of the fungicide and to develop resistance. In contrast to multi-component formulations including different active ingredients of chemical fungicides to improve protective effect, control of the pathogen in our case is achieved due to attenuation of the pathogen by an environmentally safe microbial metabolite without increasing tebuconazole or azoxystrobin concentrations. Thus, this work, showing the ability of 6-DMM to play a role in overcoming *P. nodorum* resistance to tebuconazole and azoxystrobin, confirms our hypothesis regarding the practicability of studying natural compounds attacking biochemical targets different from those by fungicides, as biological sensitizers of plant pathogens enhancing their sensitivity to fungicides.

Research on the sensitizing potential of 6-DMM *in vitro* and *in planta* will be continued. It cannot be ruled out that more effective combinations of fungicides and sensitizers will be discovered in future experiments. Such combinations might be tested against sensitive strains and resistant mutants of *P. nodorum*. In general, our current study and previously published reports clearly point to the great potential of 6-DMM as a promising agent for improving the fungicidal effectiveness of tebuconazole and azoxystrobin towards sensitive and resistant *P. nodorum*. This sensitizer could be added as a component of new formulations, to improve the fungicidal efficacy of triazoles and strobilurins towards pathogenic populations containing resistant isolates of *P. nodorum*. However, a larger number of sensitive fungal strains and resistant mutants should be included in the subsequent studies, and target mutations should be identified to evaluate the real prospects of a 6-DMM application in such populations. Contemporaneously, further research is necessary to reveal the most effective concentration combinations of the sensitizer with aforementioned fungicides, suitable for field implementation. Expanded investigations should also be carried out to assess the 6-DMM sensitizing activity towards *Zymoseptoria tritici*, another widespread leaf blotch disease-causing pathogen, which forms a common pathogenic complex within the wheat leaf canopy along with *P. nodorum*.

## Data Availability Statement

Publicly available datasets were analyzed in this study. This data can be found here: Data on 6-demethylmevinoline structure (CAS Number: 73573-88-3) are available in CAS REGISTRY^®^ database (https://www.cas.org/cas-data/cas-registry), dose rates of fungicides recommended for field treatments of wheat plants are indicated in the State Catalogue of Pesticides and Agrochemicals Approved For the Use in Russian Federation in 2022. Moscow: Ministry of Agriculture of Russian Federation (https://www.agroxxi.ru/goshandbook).

## Author Contributions

LS and VD conceived the concept of the research and designed the experiments. MK, TV, and LA performed experiments, and prepared figures and tables. KC took part in greenhouse experiments, and prepared the reagents, materials, and analysis tools. LS and VD analyzed experimental data, wrote the original draft, and reviewed and edited the drafts of the manuscript, figures, and tables. All authors reviewed and approved the manuscript.

## Funding

This study was supported by the Russian Science Foundation (project no. 18-16-00084).

## Conflict of Interest

The authors declare that the research was conducted in the absence of any commercial or financial relationships that could be construed as a potential conflict of interest.

## Publisher’s Note

All claims expressed in this article are solely those of the authors and do not necessarily represent those of their affiliated organizations, or those of the publisher, the editors and the reviewers. Any product that may be evaluated in this article, or claim that may be made by its manufacturer, is not guaranteed or endorsed by the publisher.

## Supplementary Material

Supplementary Figure 1 HPLC chromatograms of Na-salt of 6-DMM preparation obtained from *P. citrinum* 18-12 culture liquid **(A)** and the 6-DMM standard **(B)**. **(C)**, Structural formula of 6-DMM. Isolated 6-DMM was purified and quantified by HPLC (ReproSil ODS-A C18, 5µm, 250 × 131 4.0 mm, Dr. Masch GmbH, Germany) using a Waters 1525 Breeze HPLC system equipped with a Waters 2487 UV detector at 234 nm. Methanol:0.1% acetic acid (80:20 v/v) was used as the mobile phase. Prior to sensitization experiments, the 6-DMM lactone was converted to water-soluble Na-salt of 6-DMM. To do this, a preparation of 6-DMM was dissolved in hot ethanol, the ethanol solution was amended with an equimolar amount of NaOH, incubated for 30 min at gently mixing and diluted with distilled water so that the final 6-DMM content was not lower than 10% in order to prevent a pellet formation that occurred in less concentrated solutions during long storage. An insignificant difference in the retention time between 6-MMM purified form from *P. citrinum* 18-12 culture liquid (8.82 min) and the standard (9.40) is due to the fact that the analyzed sample is a Na-salt of 6-DMM, while the standard preparation is 6-DMM lactone (Sigma-Aldrich, St. Louis, MO, US).

Supplementary Table 1 The levels of minimum inhibitory concentrations (MIC)* of 6-demethylmevinolin (6-DMM) and fungicides for wild *P. nodorum* strains (M-4 and 8/B-47) as well as for tebuconazole- and azoxystrobin-resistant mutants (m8-4 and kd-18, respectively) *MIC is the lowest concentration preventing a visible colony growth. The MIC values were detected by serial dilutions according to a reference CLSI method for antifungal susceptibility testing of filamentous fungi (Espinel-Ingro et al., 2012) and also calculated using a probit analysis.
