# Supplementary material for: A Secondary Metabolite Secreted by Penicillium citrinum Is Able to Enhance Parastagonospora nodorum Sensitivity to Tebuconazole and Azoxystrobin
Source: Front Fungal Biol. 2022 Jul 7;3:889547. doi: 10.3389/ffunb.2022.889547 (PMC10512332; doi:10.3389/ffunb.2022.889547)
Supplement: Supplementary Figure 1 — HPLC chromatograms of Na-salt of 6-DMM preparation obtained from P. citrinum 18-12 culture liquid (A) and the 6-DMM standard (B). (C), Structural formula of 6-DMM. Isolated 6-DMM was purified and quantified by HPLC (ReproSil ODS-A C18, 5µm, 250 × 131 4.0 mm, Dr. Masch GmbH, Germany) using a Waters 1525 Breeze HPLC system equipped with a Waters 2487 UV detector at 234 nm. Methanol:0.1% acetic acid (80:20 v/v) was used as the mobile phase. Prior to sensitization experiments, the 6-DMM lactone was converted to water-soluble Na-salt of 6-DMM. To do this, a preparation of 6-DMM was dissolved in hot ethanol, the ethanol solution was amended with an equimolar amount of NaOH, incubated for 30 min at gently mixing and diluted with distilled water so that the final 6-DMM content was not lower than 10% in order to prevent a pellet formation that occurred in less concentrated solutions during long storage. An insignificant difference in the retention time between 6-MMM purified form from P. citrinum 18-12 culture liquid (8.82 min) and the standard (9.40) is due to the fact that the analyzed sample is a Na-salt of 6-DMM, while the standard preparation is 6-DMM lactone (Sigma-Aldrich, St. Louis, MO, US). [file DataSheet_1.zip › Supplementary Table 1.pdf]

Supplementary Table 1. The levels of minimum inhibitory concentrations (MIC)* of 6-demethylmevinolin (6-DMM) and fungicides for wild *P. nodorum* strains (M-4 and 8/B-47) as well as for tebuconazole- and azoxystrobin-resistant mutants (m8-4 and kd-18, respectively)

P. nodorum strain	MIC volume		
	6-DMM, mg/ml	Folicur®, μg/ml	Quadris®, μg/ml
M-4	5.01	12.5	100.0
m8-4	4.90	112.5	−
kd-18	5.38	−	507.5
8/B-47	5.26	32.0	−

*MIC is the lowest concentration preventing a visible colony growth. The MIC values were detected by serial dilutions according to a reference CLSI method for antifungal susceptibility testing of filamentous fungi (Espinel-Ingro et al., 2012) and also calculated using a probit analysis.
